# Upper body rate of force development and maximal strength discriminates performance levels in sport climbing

**DOI:** 10.1371/journal.pone.0249353

**Published:** 2021-03-26

**Authors:** Nicolay Stien, Vegard Albert Vereide, Atle Hole Saeterbakken, Espen Hermans, Matthew Peter Shaw, Vidar Andersen

**Affiliations:** Faculty of Education, Arts and Sports, Western Norway University of Applied Sciences, Bergen, Norway; Universita degli Studi di Milano, ITALY

## Abstract

The aim of this study was to assess and compare the maximal force and rate of force development (RFD) between intermediate, advanced and elite climbers using several different methods for calculating RFD. Fifty-seven male climbers (17 intermediate, 25 advanced, and 15 elite) performed isometric pull-ups on a climbing-specific hold while the RFD was calculated using several absolute (50, 100, 150, 200, and 250 ms from onset of force) and relative time periods (25, 50, 75, 95, and 100% of time to peak force). The maximal force was higher among elite climbers compared to advanced (ES = 1.78, p < 0.001) and intermediate climbers (ES = 1.77, p < 0.001), while no difference was observed between intermediate and advanced climbers (P = 0.898). The elite group also showed higher RFD than the other two groups at all relative time periods (ES = 1.02–1.58, p < 0.001–0.002), whereas the absolute time periods only revealed differences between the elite vs. the other groups at 50, 100 and 150 ms from the onset of force (ES = 0.72–0.84, p = 0.032–0.040). No differences in RFD were observed between the intermediate and advanced groups at any time period (p = 0.942–1.000). Maximal force and RFD, especially calculated using the longer periods of the force curve, may be used to distinguish elite climbers from advanced and intermediate climbers. The authors suggest using relative rather than absolute time periods when analyzing the RFD of climbers.

## Introduction

Sport climbing will be introduced for the first time as an Olympic sport in 2021 and has received increased attention from researchers and athletes [1]. Researchers attempting to determine which factors influence sport climbing- and bouldering-performance have identified a combination of technical [2,3], neuromuscular [4–8], anthropometric [9–11], psychological [12], and physiological factors [13]. In general, higher performing athletes are stronger than intermediate climbers, especially when climbing-specific tests and hold types are implemented [8,10,14,15]. Moreover, in previous studies examining climbers, the strength and rate of force development (RFD) of the finger flexors has also discriminated between climbing performance levels [8] and disciplines [16].

RFD is defined as the rate of the rise in force during isometric contractions, and has been used to quantify the ability to generate force rapidly [17]. When climbing harder routes, the smaller holds and more difficult moves cause a need for more force to be exerted in a shorter time window to avoid falling off the route. RFD may, therefore, be a key factor for predicting climbing performance [4,5,8], and has discriminated between skilled and international performance levels when calculated using longer time periods [8]. Previous studies have implemented a variety of testing procedures for examining RFD [8,16,18–20]. In one recent study [20], RFD was measured using a hand dynamometer, which have been shown to be less valid than specific tests (e.g., using climbing-specific holds and common climbing-positions) [21]. Conversely, Fanchini et al. [16] and Michailov et al. [19] used climbing-specific holds but isolated the finger flexors, excluding the arm- and back muscles from the testing. This might reduce the validity as, when climbing, the fingers are only responsible for maintaining contact with the holds whilst the vertical propulsive force of the climber is produced mainly by other prime movers (i.e., elbow flexors and shoulder extensors). A more promising test was used by Levernier and Laffaye [8] and Michailov et al. [19], examining maximal voluntary isometric contractions (MVIC) in a standing position. This removes constraints around the elbow and allows several prime movers to contribute to the MVIC, providing a higher validity [19]. To the authors’ best knowledge, only two studies [18,22] have assessed the RFD of the entire pulling-apparatus (finger-, arm-, shoulder- and back-muscles) in one exercise (isometric pull-ups on a climbing-specific hold). However, the authors compared climbers of different disciplines rather than performance levels.

In addition to varying test set-ups, differences in the calculation of RFD between studies limit the comparability of the findings. For example, the time periods used to calculate RFD have ranged between 150 ms to absolute RFD (RFD_100%_; calculated from the onset of force to peak force) [8,18,20,22,23]. While RFD calculated using longer time periods of the force curve may be strongly related to maximal force [24], the shorter time periods (50–250 ms) could be associated with the explosive strength required for hard and dynamic climbing moves [8,17,25], but might also be more prone to variability [8,26]. Finally, it has been suggested that RFD data should be normalized (RFD relative to maximal force) to highlight whether or not differences in RFD are caused by a difference in maximal strength alone [27,28].

While a variety of time periods have been examined in different sports and resistance exercises, no literature exists concerning which time period should be used when analyzing RFD of the entire pulling-apparatus in climbing. As varying times to reach peak force in climbing-specific tests have been observed [8,18,20], the use of relative (calculated at a given percentage of the time to reach peak force) rather than absolute time periods could be more reliable. Finally, to the authors’ best knowledge, no previous study has examined RFD among three different levels of climbers. Hence, the aim of this investigation was to assess and compare the RFD of three performance levels of climbers (intermediate, advanced, and elite), as well as examine the reliability and ability of several absolute and relative time periods to discriminate between performance levels. It was hypothesized that the advanced climbers would produce higher RFD than the intermediate climbers, and that the elite climbers would have higher RFD than the advanced and intermediate climbers. Finally, we expected RFD calculated using longer time periods of the force curve to be more reliable and more discriminatory between performance levels.

## Materials and methods

### Study design

To answer the research question, a cross-sectional between-subject comparative study was designed, including three different levels of lead climbers. To reach an adequate sample size, the data was collected over the course of two years using a standardized protocol and trained test leaders.

### Participants

Fifty-seven male amateur lead climbers volunteered for this cross-sectional study (characteristics are presented in Table 1). Participants were asked to report their climbing experience as the number of consecutive years for which they had been climbing regularly (at least one session per week). Many participants were also engaged in general resistance- or endurance training, but all included participants had climbing as their primary activity. To be included, participants had to be able to perform the experimental tests correctly (i.e., be strong enough to hang from the hold used and be able to perform a maximal-effort isometric pull-up without falling of the rung), and have a minimum self-reported climbing ability of 6b (International Rock Climbing Research Association (IRCRA) [29] = 13; intermediate level). It has been reported that self-reported climbing grades are accurate and appropriate for use in research contexts [30]. Participants also had to be without any injuries or illnesses that could limit maximal performance in the testing. None of the participants were professional climbers, but several of the included climbers were competing on a national level.

**Table 1:** Anthropometric data, climbing experience, weekly number of climbing sessions, and average onsight grade of the participants given according to the numerical grading scale (1–32) suggested by the International Rock Climbing Research Association (IRCRA). The values are presented as means (± standard deviation).

	Intermediate (n = 17)	Advanced (n = 26)	Elite (n = 14)
Age (years)	26.18 ± 1.60	29.28 ± 2.74	27.40 ± 4.29
Height (cm)	184.47 ± 12.82	179.24 ± 2.81	179.40 ± 3.33
Body mass (kg)	73.39 ± 4.21	72.09 ± 3.29	72.25 ± 3.35
Weekly sessions (n)	2.43 ± 1.22	3.20 ± 1.02^*^	4.4 ± 1.02^**^
Experience (years)	4.18 ± 1.14	6.74 ± 2.08	11.47 ± 3.44^**^
Redpoint (IRCRA)	15.82 ± 1.12	20.04 ± 0.56^⸸^	24.87 ± 0.81^⸸^‡

* = significantly greater than the intermediate group (P < 0.05).

** = significantly greater than the intermediate and advanced groups (P < 0.01).

⸸ = significantly higher Redpoint grade than the intermediate group (P < 0.01).

‡ = significantly higher Redpoint grade than the advanced group (P < 0.01).

### Ethics statement

The participants were informed verbally and in writing about the potential risks and benefits of participation and signed and informed consent form before data collection commenced. The present research procedures were in accordance with the ethical guidelines of Western Norway University of Applied Sciences, conformed to the standards of treatment of human participants in research outlined in the 5th Declaration of Helsinki, and approved by the Norwegian Centre for Research Data.

### Measurements and test procedures

Upon arrival to the laboratory, participants reported their age, climbing experience and climbing level. Their highest achieved climbing grade in the last year was reported using either the Scandinavian or French grading systems and the answers were converted to the numerical grading scale (1–32) proposed by the International Rock Climbing Research Association (IRCRA). Using the grouping system suggested by Draper et al., [29] the climbers were divided into one of the following three groups based on their self-reported maximal achieved climbing grade: intermediate group (IRCRA 10–17; n = 17), advanced group (IRCRA 18–23; n = 25), and elite group (IRCRA 24–27; n = 15). Height and body mass were then measured using a wall mounted measuring tape and a bioelectric impedance scale (Tanita MC 780MA S, Tokyo, Japan), respectively. Following the anthropometric measures, the participants performed a 15-minute light-to-moderate warm-up consisting of bouldering and traversing. The participants selected the difficulty of the boulders themselves but were instructed to avoid fatigue.

**Fig 1:**
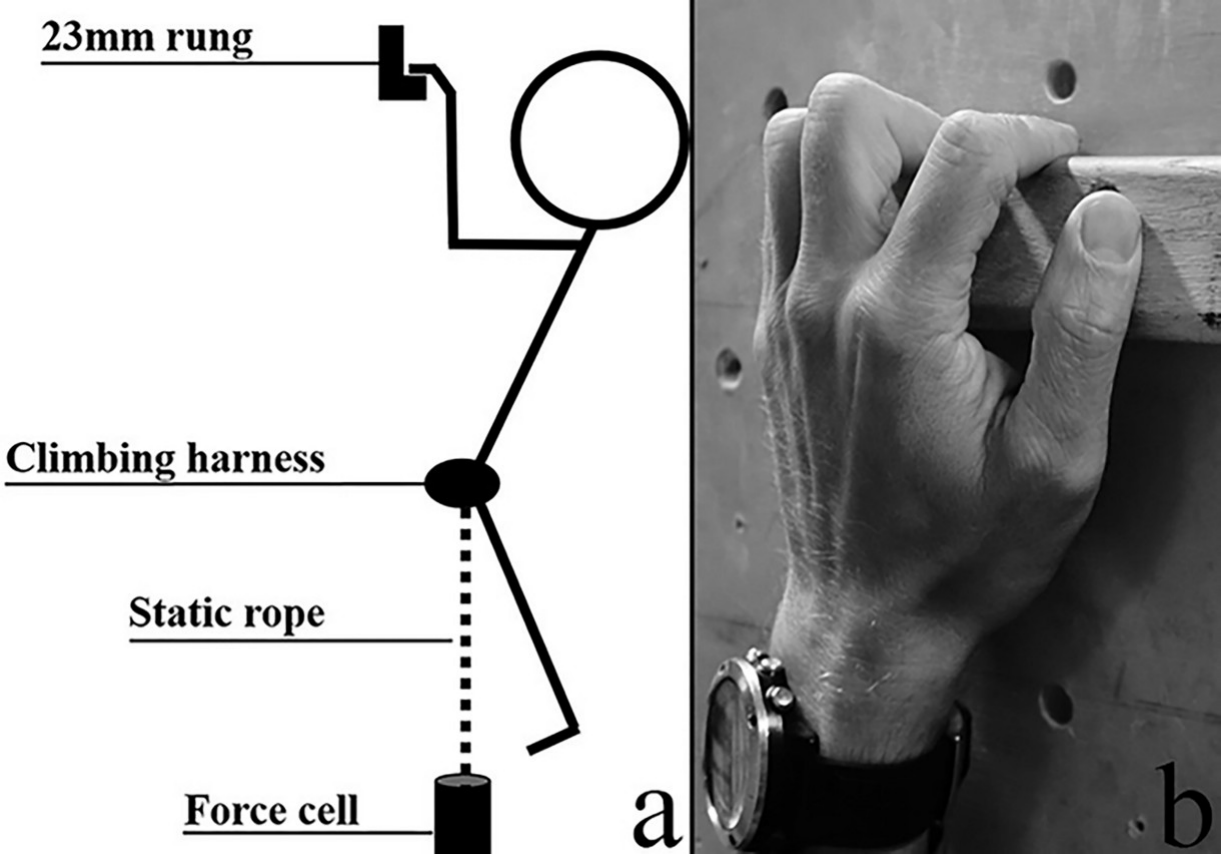
Illustration showing a) the test set-up for the isometric pull up and b) the half crimp grip on the 23mm rung.

After resting for five minutes, the experimental test began. The data was collected during an isometric pull-up performed on a 23mm deep rung (Metolius Climbing, Bend, Oregon, USA) with rounded edges using a half crimp grip with a passive thumb, self-selected width between the hands, and a 90° elbow angle [18,19,22] (Fig 1). The climbers could apply chalk (magnesium carbonate) to their hands and fingers before starting the test and the rung was regularly brushed to provide equal friction conditions for all participants. The force output was measured using a force sensor (Ergotest Innovation A/S, Porsgrunn, Norway) anchored to the ground (via an expansion bolt and hanger in the concrete floor) and attached to the participants via a static rope and the belay loop of a climbing harness positioned slightly (1–4 cm) below the iliac crest. The length of the rope was adjusted for each participant to achieve the correct elbow angle (measured with goniometer) and the placement of the harness was controlled between attempts.

Following verbal instructions, participants pulled themselves up to a 90° elbow angle (where the rope became taut) and maintained the position for approximately one second. They had to hang still (no more than ± 5N fluctuation in force for 1000 ms) before the contraction and an attempt was annulled if a dip in the force (small countermovement creating slack in the rope) was observed prior to the onset of force. The participants were then verbally encouraged to perform an isometric pull-up as quickly and forceful as possible [28] and maintain maximal force for three-to-five seconds. For an attempt to be correctly executed, the force had to increase continually, without a plateau, to the peak force output (peak force coefficient of variation (CV) = 12.9%, 9.2%, and 9.1% for the intermediate, advanced and elite groups, respectively). As the participants were experienced climbers, no familiarization session was implemented, but three attempts were given to ensure that optimal performance was reached. The best result was used in the between-groups analyses, while all three attempts were registered to calculate intra-class correlations (ICC) and CV within-participants during one testing session. The attempt with the steepest force curve (highest absolute RFD from onset to peak force) was considered the best attempt. Participants were shown the force curve after each attempt and given feedback on how to improve for their next attempt. Three minutes of rest were given between attempts [8].

The force output was recorded by the force sensor at 200Hz and analyzed using commercial software (MuscleLab v. 10.4.37.4073, Ergotest Innovation A/S, Porsgrunn, Norway). The onset of contraction was identified visually, which has been proposed as more sensitive and accurate than automated detection [28]. The onset was determined at the point when the force increased more than 5 N from the baseline over a 5 ms window. This manual method has been shown to be reliable [25] and has been previously implemented in similar investigations [8,20]. The baseline force could not exceed 100 N and the mean baseline force across all attempts was 58 ± 36N. The same researcher analyzed all the data to avoid inter-rater variability in determination of the onset. The RFD was collected from the recorded force curves at different time periods (0–50, 0–100, 0–150, 0–200 and 0–250 ms) from the onset of force [31]. Peak force (N) and the time to reach peak force (ms) was also registered in order to calculate relative time periods (25%, 50%, 75%, 95% and 100%) from the onset of contraction. Finally, the RFD_100%_ was normalized to the peak force to investigate what influence the maximal strength had on the potential differences in RFD between groups. Every force curve was strictly evaluated before inclusion in the analyses.

### Statistics

Kolmogorov-Smirnov test and visual inspection of the QQ-plots showed normally distributed data (P = 0.053–0.200). SPSS statistical software (Version 25.0, SPSS Inc., Chicago, IL, USA) was used for all analyses. The reliability of each RFD measure was assessed using ICC and CV ((population standard deviation / population mean) × 100). ICC values less than 0.5, between 0.5 and 0.75, between 0.75 and 0.9, and greater than 0.9 were classified as poor, moderate, good, and excellent, respectively [32]. CV values less than 10% were considered acceptable [8]. A one-way analysis of variance (ANOVA) was used to examine whether there were differences between the three groups for the tested variables. When significant differences were detected, Bonferroni post-hoc tests were used to identify where the differences lay. The alpha level was set at 0.05 for statistical significance. The Cohen’s d effect sizes (ES) for the differences between the groups were calculated as the means divided by the pooled standard deviation. An ES of < 0.2 was considered trivial, > 0.2 small, > 0.5 moderate, and > 0.8 large [33].

## Results

### Reliability

The ICC and CV between the three attempts using the relative and absolute time periods are presented in Table 2A and 2B, respectively. The ICCs ranged from moderate to excellent for all levels of climbers. All CV values for the intermediate and advanced climbers were unacceptable (CV = 16.9 to 31.3%), whereas the CV values for the elite climbers were acceptable (≤ 10%) only when using the entire force curve (RFD_100_) and 250 ms from the onset.

**Table 2:** a. The mean rate of force development (Newton × s^-1^) from the three attempts for each group using the relative time periods with the mean rate of force development and the intra-class correlation (ICC) and coefficient of variation (CV) between the three attempts. b. The mean rate of force development (Newton × s^-1^) from the three attempts for each group using the absolute time periods with the mean rate of force development and the intra-class correlation (ICC) and coefficient of variation (CV) between the three attempts.

**Intermediate group**			
Time period	Mean RFD	CV (%)	ICC
25%	610 ± 274	27.8 ± 15.3	0.798
50%	948 ± 443	21.1 ± 12.1	0.914
75%	1214 ± 563	21.0 ± 14.2	0.905
95%	1209 ± 571	20.3 ± 11.7	0.916
100%	1166 ± 549	20.0 ± 11.3	0.918
**Advanced group**			
Time period	Mean RFD	CV (%)	ICC
25%	762 ± 361	28.2 ± 14.7	0.857
50%	1084 ± 402	16.9 ± 12.2	0.817
75%	1342 ± 472	18.0 ± 11.3	0.830
95%	1311 ± 473	18.7 ± 10.6	0.858
100%	1272 ± 468	17.9 ± 9.5	0.877
**Elite group**			
Time period	Mean RFD	CV (%)	ICC
25%	1450 ± 740	19.7 ± 15.4	0.763
50%	2259 ± 1224	13.9 ± 13.6	0.817
75%	2662 ± 1074	10.6 ± 7.9	0.971
95%	2590 ± 1030	10.7 ± 6.5	0.985
100%	2519 ± 978	8.9 ± 5.2	0.985
**Intermediate group**			
Time period	Mean RFD	CV (%)	ICC
50ms	628 ± 490	27.7 ± 19.2	0.921
100ms	906 ± 745	24.5 ± 13.9	0.957
150ms	1154 ± 829	23.4 ± 16.9	0.955
200ms	1185 ± 585	24.4 ± 16.9	0.850
250ms	1107 ± 469	26.0 ± 14.9	0.744
**Advanced group**			
Time period	Mean RFD	CV %	ICC
50ms	626 ± 260	31.3 ± 15.9	0.687
100ms	917 ± 471	30.1 ± 17.1	0.776
150ms	1197 ± 604	25.9 ± 18.0	0.691
200ms	1364 ± 635	23.0 ± 17.6	0.746
250ms	1222 ± 490	18.9 ± 12.1	0.860
**Elite group**			
Time period	Mean RFD	CV %	ICC
50ms	1180 ± 915	26.7 ± 24.1	0.802
100ms	1692 ± 1161	18.6 ± 15.1	0.934
150ms	2065 ± 1084	11.5 ± 9.7	0.986
200ms	1943 ± 778	11.4 ± 9.6	0.956
250ms	1547 ± 604	10.0 ± 8.6	0.966

RFD and CV values are presented with mean ± standard deviation.

### Baseline results

No differences in anthropometric variables between the three groups of climbers were detected (p = 0.272–0.852). No significant difference in climbing experience was found between the intermediate and advanced climbers (p = 0.279), while the elite group had a longer experience than the intermediate (ES = 1.74, p < 0.001) and advanced climbers (ES = 0.86, p = 0.006; See Table 1).

### RFD between groups

Significant differences in RFD were found at all relative time periods (F = 10.197–16.631, all p < 0.001). Post hoc tests revealed no differences between the intermediate and advanced groups (p = 0.942–1.000, while the elite group had higher RFD than both the intermediate and advanced climbers at all measures (p < 0.001–0.002; Table 3).

**Table 3:** The rate of force development (Newton × s^-1^) from the best attempt for the elite intermediate (IG), advanced (AG) and elite groups (EG) and the effect sizes for the between groups differences.

Time Period	Intermediate group	Advanced group	Elite group	Effect size IG *vs*. AG	Effect size AG *vs*. EG	Effect size EG *vs*. IG
50ms	801 ± 625	799 ± 338	1457 ± 1260	0.00	0.82^*^	0.70
100ms	1069 ± 837	1146 ± 588	1928 ± 1364	0.11	0.80^*^	0.78^*^
150ms	1364 ± 917	1495 ± 862	2236 ± 1154	0.15	0.73	0.84^*^
200ms	1456 ± 749	1664 ± 852	2117 ± 873	0.26	0.52	0.82
250ms	1405 ± 713	1423 ± 605	1675 ± 671	0.03	0.39	0.39
25%	747 ± 332	952 ± 439	1719 ± 1065	0.53	1.02^⸸^	1.39^⸸^
50%	1092 ± 477	1272 ± 510	2555 ± 1699	0.36	1.16^⸸^	1.34^⸸^
75%	1426 ± 664	1562 ± 559	2889 ± 1196	0.22	1.51^⸸^	1.57^⸸^
95%	1426 ± 689	1548 ± 569	2807 ± 1085	0.19	1.52^⸸^	1.56^⸸^
100%	1372 ± 662	1492 ± 562	2709 ± 1051	0.20	1.51^⸸^	1.56^⸸^

All values are presented with mean ± standard deviation.

* = significant difference at the P < 0.05 level.

⸸ = significant difference at the P < 0.01 level.

For the absolute time periods, the analyses revealed significant differences between the groups for RFD_50_ (F = 4.128, p = 0.021), RFD_100_ (F = 4.368, p = 0.017), and RFD_150_ (F = 3.853, p = 0.027), but not for RFD_200_ (F = 2.362, p = 0.104) or RFD_250_ (F = 0.504, p = 0.608). No differences were found between the intermediate and advanced groups (p = 1.000). The elite climbers produced higher RFD than the intermediate group at RFD_100_ (p = 0.032) and RFD_150_ (p = 0.040), and higher RFD than the advanced group at RFD_50_ (p = 0.032) and RFD_100_ (p = 0.035; Table 3).

### Force and time

**Fig 2:**
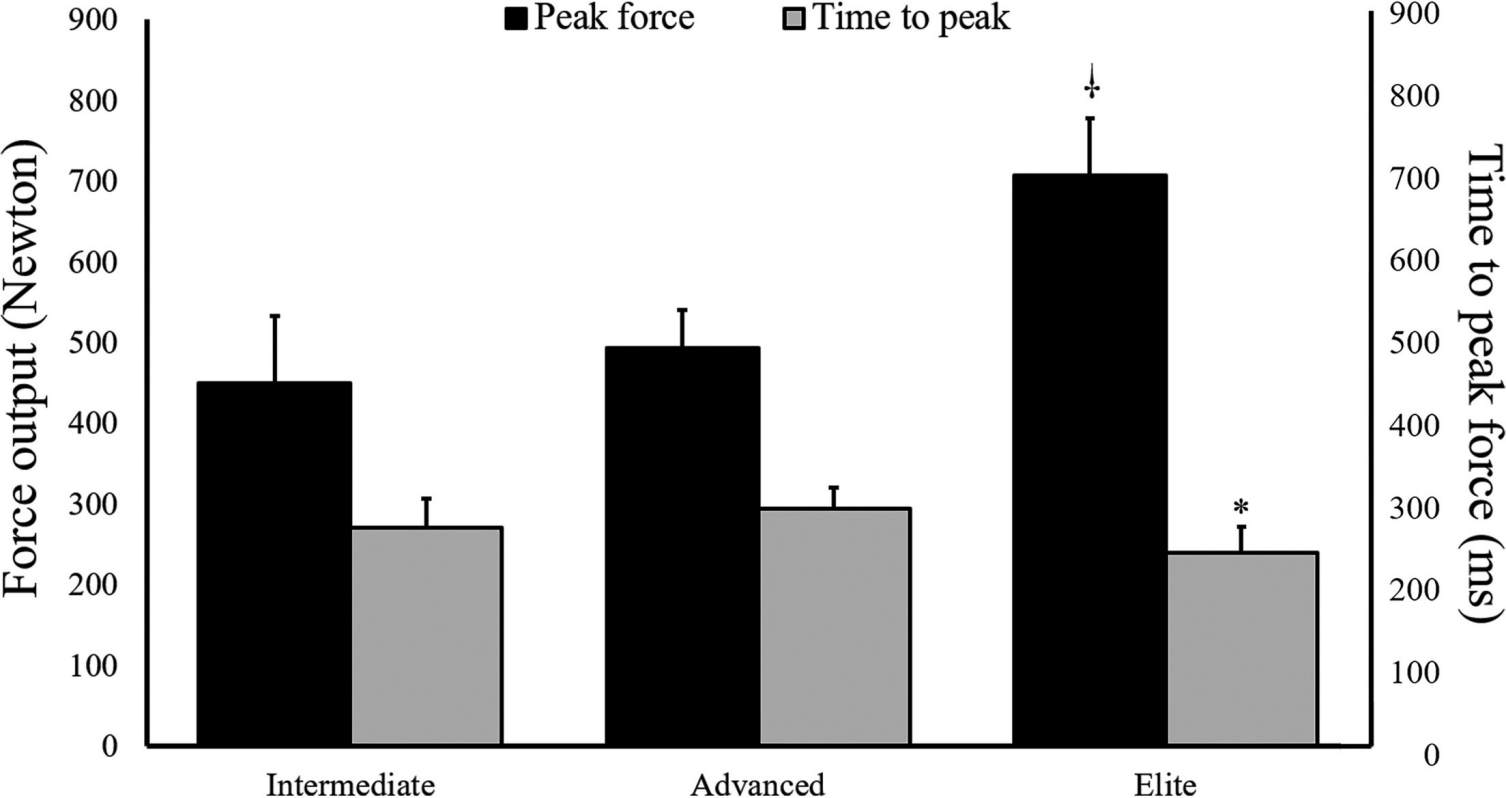
The maximal force output (Newton) and the time to reach peak force (milliseconds) for the three groups. ⸸ = significantly higher than the intermediate and advanced groups (P < 0.01). * = significantly lower than the advanced group (P < 0.01).

To investigate the relative contribution of the two quotients of RFD, the peak force and the time to reach peak force were analyzed. Differences between the groups were found for the time- (F = 3.377, p = 0.041) and force-factors (F = 16.932, p < 0.001). Post hoc tests showed that the intermediate group did not differ from the advanced (p = 0.767) or elite groups (p = 0.526) in the time to reach peak force, while the elite group reached peak force faster than the advanced climbers (ES = 0.88, p = 0.036; Fig 2). The elite group produced higher peak force output than the intermediate (ES = 1.77, p < 0.001) and advanced groups (ES = 1.78, p < 0.001), while no significant difference was found between the intermediate and advanced groups (p = 0.898; Fig 2).

### Normalized RFD

Finally, the RFD relative to the peak force was significantly different between groups (F = 4.301, p = 0.018). There was no difference between the intermediate and advanced groups (p = 0.855), while the elite group achieved a higher normalized RFD than both the intermediate (ES = 0.87, p = 0.020) and advanced groups (ES = 0.80, p = 0.017).

## Discussion

The aim of this study was to examine the ability of different RFD measures to discriminate between performance levels among climbers. In line with the primary hypothesis, the elite climbers produced higher RFD than the intermediate and advanced climbers. Conversely, no significant differences were found between the intermediate and advanced climbers. Based on these findings, RFD may not be a crucial component for climbing performance before reaching the more demanding grades (> 24 IRCRA). Whereas the shift from an intermediate to an advanced climber may be implemented by practicing other factors such as endurance, technical skills or psychological factors [2,3,13], a progression to the elite level seems to entail a prominent improvement in RFD.

The higher RFD produced by the elite climbers was accompanied by a notably higher peak force output than the other groups, while the time to reach peak force was only lower than the advanced climbers. Several years of climbing hard moves on shallow holds has likely produced a training stimulus for promoting maximal strength and RFD of the finger flexors and pulling apparatus. As the peak force output provided much clearer differences between the groups than the time to reach peak force, one could assume that using longer times from the onset of force would be better suited for distinguishing between performance levels. Indeed, it has been reported that RFD calculated using longer times from onset of force is more strongly related to maximal force [24]. Importantly, the RFD in the elite group was still greater than in the intermediate and advanced groups following normalization. Hence, the higher peak force alone did not cause the differences in RFD. However, it should be noted that the ES for the differences were reduced following normalization, suggesting that a meaningful portion of the differences in RFD is caused by the higher peak force output in the elite group. Since advanced and intermediate climbers possess less climbing-specific strength of the finger flexors than the elites, performing a maximal-effort contraction using the shallow rung might limit the RFD substantially [18]. This could reduce the potential differences between these two groups while enhancing the difference between the non-elite groups and the elite group.

In contrast to the hypothesis, the late phase of the absolute time periods did not produce more distinctive differences between the groups than the early phase. Conversely, differences between groups were only significant using the 50 ms, 100 ms and 150 ms absolute time periods, and the between-groups difference effect sizes were notably lower using the 200 ms and 250 ms time periods. One potential explanation could be that maximal strength accounts for less of the difference than neurological adaptations to years of attempting hard routes that require rapid force production [28]. In contrast to the absolute measures, the relative measures produced both lower CV values and more distinct between-groups differences, especially when examining the longer durations from the onset of force. As previously speculated [8], the maximal number of muscle fibers recruited while exerting maximal force is likely more reproduceable than the time taken to recruit the fibers. As large variations between individuals’ times to reach peak force were observed in this (150 to 730 ms) and previous studies (~ 400 to 1000 ms) [8,18,22], using relative time periods should be the preferred method when examining the entire pulling-apparatus of climbers. For example, if an individual uses ≥ 500 ms to reach peak force, the longest absolute time period (250 ms) would still represent the earlier phase of the force curve. Hence, relative time periods could be more practically applicable than the traditional division of early and late phases [28] in tasks typically requiring longer than 250 ms to reach peak force.

Examining the remaining relative (RFD_50%_—RFD_100%_) and absolute measures (RFD_100_—RFD_250_), the intermediate and advanced climbers produced notably higher CV values (16.9–30.1%) than the elite group (8.9–19.7%). These findings are in agreement with those of Levernier and Laffaye [8] who proposed that increasing skill level could be associated with an improved ability to reproduce similar force outputs across several attempts. More climbing experience probably also produces a more efficient reqruitment of the available motor units [34], thereby allowing for a more rapid force production across attempts. Although physiological differences likely account for the difference between the elite and the non-elite climbers (intermediate and advanced), lack of differences between the intermediate and advanced group could partly be explained by the unacceptable CV values observed for these groups. Finally, the results could potentially be explained by the small difference in climbing experience between the intermediate and advanced groups, as well as the fact that the intermediate climbers had a self-reported redpoint grade of 15.82 (IRCRA suggests intermediate classification between 10 and 17) [29]. Hence, the participants in the intermediate groups could be described as higher intermediate.

For the elite group, lower CV values were found when calculating RFD using the relative measures compared to the absolute measures. The higher reliability using the relative time periods demonstrates that practitioners can be more confident that differences in data are true differences. As only the RFD_100%_ and RFD_250_ produced good CV values (≤ 10%), results obtained using the remaining time periods should be interpreted with caution. When investigating the intra-class correlations of the climbing-specific test used in this study, moderate-to-excellent reliability was found for the absolute measures, and good-to-excellent reliability for the relative measures. Hence, RFD measured in the current test set-up was consistent across three attempts, especially when using ≥75% of the force curve (ICC = 0.830–0.985). Importantly, the reliability of the measurements improved when increasing the time periods used to calculate RFD, suggesting that the early phase should not be used during the current and similar test set-ups. Importantly, testing the entire pulling-apparatus provides a highly climbing-specific task, but allows for more variation between attempts than when testing muscles isolated (e.g., with the elbow constrained). Researchers must, therefore, consider a potential trade-off between reliability and validity when selecting testing procedures. Finally, based on the observed CV results and lack of differences between the intermediate and advanced groups, the high finger-strength demands of performing a maximal-effort isometric pull-up on a shallow rung may be better suited for examining elite climbers. Although the current test set-up proved useful for discriminating elite climbers from the advanced and intermediate groups, the observed variation indicate that the test may be unreliable for detecting changes in RFD on an individual level. Interestingly, the current multi-joint isometric testing produced CV values (8.9–28.2% for RFD_100_) that were similar to what has been reported during isolated finger flexor RFD testing (7.8–28.3%) [8].

Although the present findings provide a new insight to the use of RFD when monitoring climbers, the study had some limitations that should be considered when interpreting the results. Importantly, only male climbers were included in this study and the findings might not necessarily be generalizable to female climbers at the same level. Furthermore, no familiarization session was performed as it was expected that experienced climbers would be able to perform the test adequately. Still, several attempts were given to ensure that maximal performance was reached. Importantly, it should be noted that different climbing test set-ups (e.g., isolated finger flexor tests, climbing-specific holds vs. dynamometers, seated position, etc.) likely produce different force curves, which makes it problematic to suggest a general recommendation for calculating RFD. For example, the isometric test used in the present study produces a distinctive force peak, not reported in studies testing the finger flexors in isolation [20] or near isolation [8]. The peak likely occurs due to elastic components within several muscle groups [35], and a slight shift of the body caused by alterations of the shoulder and elbow joints when applying maximal force. Still, the peak is consistent across attempts (mean CV = 10.3%). The low sampling rate (200 Hz) could potentially challenge the reliability of the results as it makes it difficult to perfectly identify of the onset and peak forces. However, using the 5N threshold for onset determination, 200Hz resolution can accurately identify the onset (to the closest 5ms). Finally, as isometric testing is not technically difficult, different strategies or focus (e.g., trying to gain a high peak force output rather than RFD) could result in a meaningful change in force output. Therefore, a familiarization session could have improved the reliability and should be included in future studies.

The current study adds to the growing body of literature describing climbers of different performance levels and identifying RFD as a key discriminatory factor. To the authors’ best knowledge, this was the first study to compare three levels of climbers and demonstrate the meaningful differences in RFD between elite level and non-elite (advanced and intermediate) climbers. In comparison, Levernier et al. [8] also included three different groups, but one of the groups included non-climbers. Furthermore, although RFD has been suggested by many as a dependable method for assessing and classifying climbers [8,16,18,20], the methodological approaches vary between studies. For example, Levernier et al. [8] tested RFD unilaterally while standing on the ground, which mimics actual climbing to a lesser degree than the test used in our study. In line with our findings, the authors reported significant differences in RFD between performance levels. However, Levernier et al. [8] identified a varying ability of detecting between-levels differences between different absolute and relative time periods (50, 100, and 200 ms from onset, as well as 95% of max force). This provided a rationale for examining several measures of RFD and to assess which measure was the most discriminatory between climbers of different levels. Based on the present findings and the observed differences in the time to develop maximal force, we suggest using the longer (≥75% of the time to peak force) and relative time periods rather than absolute time periods when assessing climbers. Importantly, and in agreement with previous findings [8,26], the CV values using the shortest durations from the onset of force were the least reliable measurements and may, therefore, not indicate true differences. Conversely, as differences were detected using the 50 ms, 100 ms and 150 ms time periods despite the high CV values, one could speculate that the actual difference between levels are particularly prominent during the early phase of force production. Researchers should consider the present findings when designing studies for monitoring climbers. Future research should examine the validity to climbing when using different time periods during demanding climbing specific tests. Finally, when testing the entire pulling-apparatus, video motion analysis could be a useful tool for detecting variations in technical execution.

## Conclusion

No differences were found between the intermediate and advanced climbers, but the elite group reached a distinctly higher RFD and force output than both the other groups. Advancing through the lower climbing grades may be achieved by improving characteristics not measured in the present study (e.g., mental or technical skills), whereas a progression to the elite grades (> 24 IRCRA) appears to entail a marked increase in RFD and maximal force. The lack of differences in force and RFD between the intermediate and advanced climbers could be explained by the small differences in climbing experience and red-point grade, as well as the higher variability in results observed in these groups compared to the elite group. As lower variability was observed in the elite group, the present test set-up may be better suiter for examining elite climbers than non-elite climbers.

## Supporting information

S1 Dataset (XLSX)
